# Diffusion tensor imaging and diffusion kurtosis imaging of the pancreas - feasibility, robustness and protocol comparison in a healthy population

**DOI:** 10.1007/s00261-025-04889-w

**Published:** 2025-03-26

**Authors:** Carlos Bilreiro, Luisa Andrade, Rafael Henriques, Nuno Loução, Celso Matos, Noam Shemesh

**Affiliations:** 1 Champalimaud Foundation, Lisbon, Portugal; 2 Universidade Nova de Lisboa, Lisbon, Portugal; 3 CUF Tejo Hospital, Lisbon, Portugal

**Keywords:** Pancreas, Diffusion tensor imaging, Diffusion kurtosis imaging, Abdomen, Feasibility studies, Healthy volunteers

## Abstract

**Purpose:**

This study aims to determine the feasibility, image quality, intra-subject repeatability and inter-reader variability of Diffusion tensor (DTI) and Diffusion kurtosis imaging (DKI) for pancreatic imaging using different protocols and report normative values in healthy individuals.

**Methods:**

Single-institution prospective study performed on healthy volunteers in a clinical 3T scanner, using two different protocols (6/16 diffusion directions). Acquisitions were repeated twice to assess intra-subject repeatability. To assess inter-reader variability, Mean diffusivity (MD), Axial diffusivity (AD), Radial diffusivity (RD), Apparent diffusion coefficient (ADC) and Mean kurtosis (MK) values were extracted from segmented pancreas by two radiologists. A Likert scale was used by both readers to assess subjective image quality.

**Results:**

Twelve healthy volunteers were recruited for each MRI protocol. The 6 diffusion directions protocol was shorter: 7 min vs. 14 min (corresponding to 4 min vs. 7.5 min for a DTI only reconstruction). No differences in image quality were found between protocols. Only MK maps showed implausible estimates, leading to the exclusion of median 16% and 17.7% pixels for the 6- and 16-direction protocols, respectively. Intra-subject repeatability was determined with negligible coefficients of repeatability for DTI; however, MK presented slightly higher values. Inter-reader agreement was excellent for all maps (ICC > 0.9).

**Conclusions:**

DTI and DKI of the pancreas are feasible in clinical settings, with excellent inter-observer agreement and good image quality. Intra-subject repeatability is excellent for DTI, but some variability was observed with DKI. A 6-directions protocol may be preferred due to faster acquisition without quantitatively compromising estimates. MK inaccuracies prompt further research for improving artifact correction.

**Graphical abstract:**

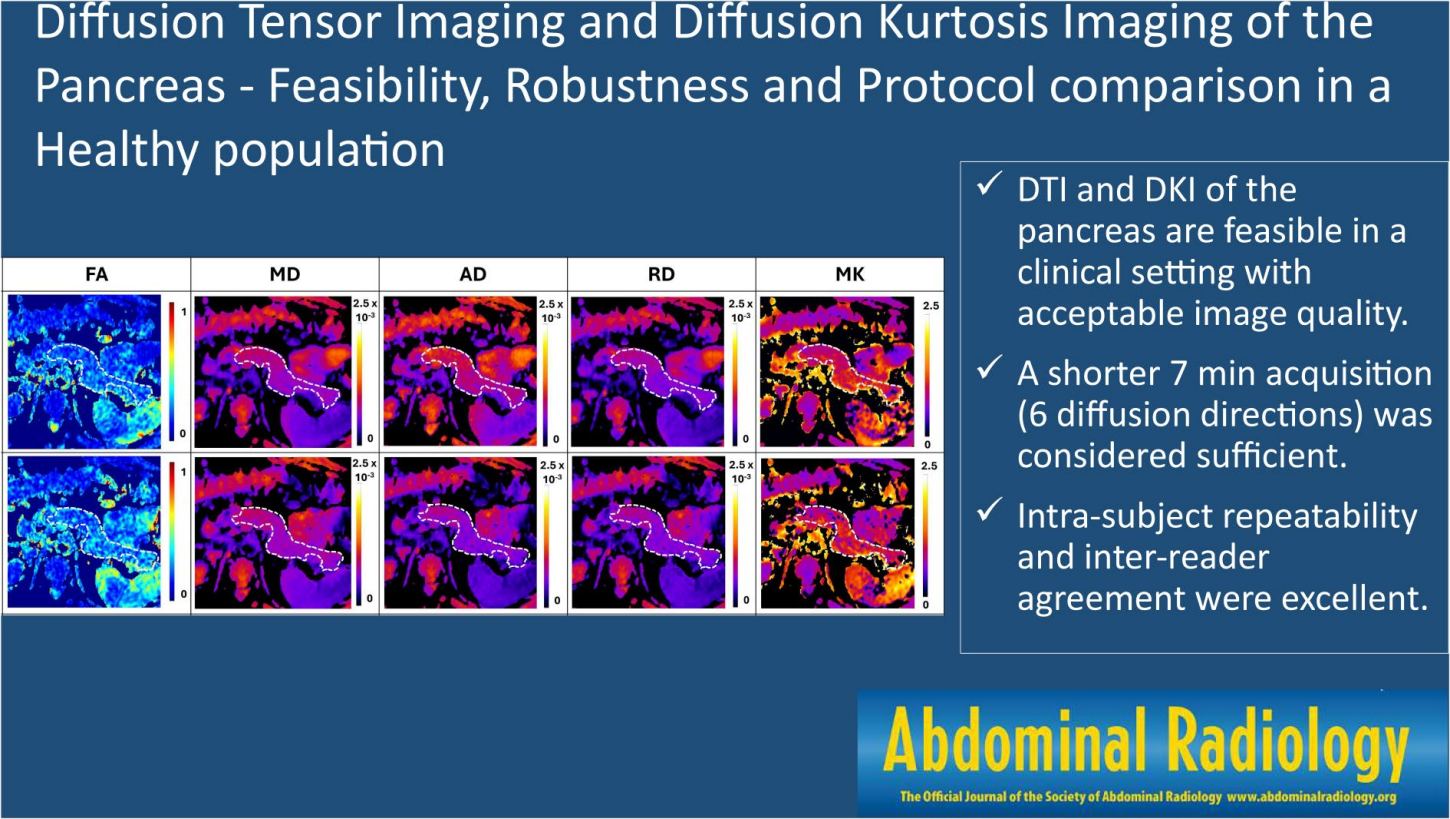

**Supplementary Information:**

The online version contains supplementary material available at 10.1007/s00261-025-04889-w.

## Background

Technological advances in recent years have provided useful tools for pancreatic imaging, in both malignant and benign pathological processes. MRI with diffusion-weighted imaging (DWI) has high sensitivity for detecting liver metastases, neuroendocrine tumors, pancreatic ductal adenocarcinoma (PDAC), auto-immune pancreatitis, and can also help differentiate mass-forming pancreatitis from PDAC [1–5]. However, several issues remain poorly addressed by current pancreatic imaging, including characterization and risk stratification of side-branch intraductal papillary mucinous neoplasms (SB-IPMN), early detection of PDAC and precursor lesions, prognostication of patients with PDAC and neuroendocrine tumors, and assessing response after neoadjuvant therapy [6–11].

DWI’s correlation with cellular density is widely explored in pancreatic imaging with the use of Apparent Diffusion Coefficients (ADC) [12]. However, ADC is limited in capturing the full complexity of tissue microstructure, as it does not account for the directional dependence of diffusion, i.e. anisotropy. Diffusion-tensor imaging (DTI) is mostly used in neuroimaging applications, providing a rotationally invariant representation of the diffusion process and quantifying diffusion anisotropy [13]. Another aspect of the diffusion process not captured by ADC is the non-Gaussian behavior of diffusion, which has been associated with microstructural heterogeneity and can be quantified with Diffusion Kurtosis Imaging (DKI) [13–15]. Previous studies have tested DTI on patients with pancreatic cancer and acute pancreatitis, and DKI in patients with pancreatic cancer, providing encouraging data for these techniques as sources of non-invasive biomarkers [16–18]. Furthermore, a recent pre-clinical study reported DTI to have clear diagnostic potential for pancreatic pre-malignant lesions– pancreatic intraepithelial neoplasia [19]. However, DTI and DKI have technically demanding acquisitions (large number of diffusion directions, high b values), and optimal acquisition parameters for pancreatic DTI/DKI are still unclear. This entails lack of standardization in acquisition protocols and consequential variability in quantitative estimates across different studies, while also hindering the development of clinical applications [16–18, 20]. Additionally, DTI and DKI’s clinical feasibility as a single acquisition, as well as their robustness in the clinical setting and image quality assessed by radiologists are still unknown.

Considering the potential of DTI and DKI for pancreatic imaging, the aim of this study is to determine the feasibility, robustness and inter-reader variability of these techniques for pancreatic imaging in a clinical setting. We compared different acquisition protocols with different number of diffusion directions and acquisition lengths, and described the expected findings in a healthy population, providing a clinically feasible short acquisition protocol and data on pancreatic DTI and DKI’s image quality and robustness.

## Methods

This single-institution prospective study with healthy volunteers received approval from Champalimaud Foundation Ethics Committee (project “DWI-P”), and each participant gave a fully informed consent before undergoing MRI.

### Participants

Healthy volunteers were recruited from the Institution’s staff and in an opportunistic way from visitors to the Institution.

Inclusion criteria were people aged 18 to 65 years-old, healthy, willing to participate and undergo MRI.

Volunteers with history of any pancreatic disease (acute or chronic pancreatitis, auto-immune pancreatitis, pancreatic exocrine insufficiency, Diabetes Mellitus, pancreatic tumors) were excluded from the study. Volunteers with contraindications for MRI, including claustrophobia or incompatible medical devices, were also excluded from the study.

### MRI Acquisition

Each participant underwent MRI in a clinical 3T scanner (Ingenia^®^, Philips NV, The Netherlands) with a 16-channel phased-array body coil. Participants were instructed to fast for at least 4 h before MRI.

The acquisition included a T2-weighted Turbo Spin-echo (TSE) in the axial plane, for pancreatic anatomical definition. For DTI and DKI, a 2D Spin-echo Echo-planar-imaging (SE-EPI) was acquired with 6 slices encompassing the entire pancreas, with the following b values: 0, 200, 1000 and 1700 s/mm^2^ [21]. The complete parameter list is found in Table 1.

**Table 1:** MRI pulse sequence parameters

Image acquisition	T2-weighted	DTI	DKI
Magnetic field strength	3 T	3 T	3 T
Pulse sequence	TSE	SE-EPI	SE-EPI
In-plane resolution (mm)	3 × 3	3 × 3	3 × 3
Slice thickness (mm)	3	3	3
Number of slices	30	6	6
Slice gap (mm)	3	10	10
TR	1207	2600	2600
TE	80	90	90
Signal averages	1	2 (b200), 4 (b1000)	2 (b200), 4 (b1000), 5 (b1700)
Acceleration factor	2	-	-
Partial-Fourier reduction factor	1	1.53	1.53
Fat-suppression	No	SPIR	SPIR
Parallel acquisition	SENSE (*R* = 2)	SENSE (*R* = 2)	SENSE (*R* = 2)
B values (s/mm^2^)	-	200, 1000	200, 1000, 1700
Diffusion directions	-	6 / 16	6 / 16
Respiratory triggering	Yes	Yes	Yes

DTI: Diffusion Tensor Imaging; DKI: Diffusion Kurtosis Imaging; TE: Echo time; TR: Repetition time; R: parallel acquisition acceleration factor; TSE: Turbo spin-echo; SE-EPI: Spin-echo Echo-planar imaging; SENSE: Sensitivity Encoding; SPIR: Spectral Presaturation with Inversion recovery

Two different diffusion direction protocols were tested: 16 and 6 evenly diffusion directions. The first protocol with 16 directions was aimed at providing a more robust representation of the diffusion tensor, while also providing redundant data for a more effective denoising, while sacrificing acquisition time length [22–24]. The second protocol with 6 directions provided a significantly faster acquisition, however with the minimum number of directions required for estimating the diffusion tensor, and with less redundant data for the denoising algorithm [23]. Scan lengths were recorded for comparison, as acquisition time was variable due to respiratory triggering.

Acquisitions were repeated twice in the same scanning session, in order to assess intra-subject repeatability.

### Image processing

Datasets were analyzed using in-house developed code in MATLAB™ (MathWorks Inc., Natick, MA). Pre-processing included denoising based on Marchenko-Pastur principal component analysis (MP-PCA) and Gibbs unringing algorithms [24, 25].

DTI was fitted per voxel with a weighted-least-squares solution as described by Veraart, et al., using the acquired b values of 200 and 1000 s/mm^2^ for removing perfusion-related effects [21, 26, 27]. This first calculation provides for each voxel a six-parameter tensor, from which three principal eigenvectors can be extracted, each with a corresponding eigenvalue. These eigenvalues (λ_1_, λ_2_, λ_3_) numbered from largest to lowest, represent the three orthogonal diffusion coefficients in the diffusion frame of each voxel. From these diffusion coefficients, mean diffusivity (MD), axial diffusivity (AD), radial diffusivity (RD) and fractional anisotropy (FA) maps were produced, for each acquisition of each volunteer. MD was calculated as the average diffusion coefficient, as follows:

$$\:MD=\:\frac{{{\uplambda\:}}_{1}+\:{{\uplambda\:}}_{2}+\:{{\uplambda\:}}_{3}}{3}$$

AD, the largest diffusion coefficient of each voxel was equal to λ_1_. RD was calculated as the average of the two smaller diffusion coefficients:

$$\:RD=\:\frac{{{\uplambda\:}}_{2}+{{\uplambda\:}}_{3}}{2}$$

FA, quantifying the amount of diffusion anisotropy in each voxel, was calculated as previously described [28]:

$$FA = \sqrt {\begin{array}{*{20}{c}}{3/2\left( {{{\left( {{\lambda _2} - MD} \right)}^2} + {{\left( {{\lambda _2} - MD} \right)}^2}} \right.} \\{\left. {+ {{\left( {{\lambda _3} - MD} \right)}^2}} \right)/\left( {{\lambda _1}^2 + {\lambda _2}^2 + {\lambda _3}^2} \right)}\end{array}} $$

DKI was fitted to the directional averaged acquired data using b values of 200, 1000 and 1700 s/mm^2^. The full details for DKI fitting in directional averaged signals are described in [29, 30], however, in short, mean signals for each b value are computed as the average from all directions before fitting. Then, the DKI model is fitted using a weighted linear least squares solution of the following expression:

$$\:MS\left(b\right)\approx\:{S}_{0}{e}^{(-bMD+\frac{1}{6}{b}^{2}{MD}^{2}MK)}$$

Here, MS represents the directional averaged diffusion-weighted signal for each b value, S_0_ the non-diffusion-weighted signal, MD is the mean diffusivity and MK the mean kurtosis. MK maps were then produced for analysis, for each acquisition for each volunteer.

ADC maps were produced for comparison as in standard clinical practice, using a monoexponential fit with b values of 200 and 1000 s/mm^2^:

$$\:S\left(b\right)={S}_{0}{e}^{(-bADC)}$$

Where S represents the measured diffusion-weighted signal for a given direction. Only the first diffusion direction was used to calculate ADC, as in clinical practice, following the isotropic diffusion principle assumed by the conventional unidirectional model of DWI.

### Image Analysis

Two abdominal radiologists, with 11 and 13 years of experience, delineated the entire pancreas in each slice using MATLAB™’s built-in segmentation tools, on b = 0 s/mm^2^ images and supported by the T2-weighted images. The resulting Regions of interest (ROI) were then placed in the corresponding ADC, FA, MD, AD, RD and MK maps, for extracting quantitative data.

Measurements from each reader were compared to assess inter-reader variability. Measurements from each volunteer, regarding the two repeated acquisitions, were compared to assess intra-subject repeatability.

The results from each protocol (16 and 6 diffusion directions) were compared for each map. Besides median values and range comparisons, the 90th and 10th percentiles of the DTI, DKI and ADC maps were also extracted from each participant, in order to provide a more granular analysis of extreme measurements. Finally, a measurement of excluded voxels with implausible values due to artifactual results (i.e. negative diffusivity/kurtosis values, kurtosis values larger than 2.5, FA and diffusivity values larger than 1) was compared between each protocol for each map to assess if any of the protocols was more robust in terms of artifactual measurements. The > 2.5 cutoff for implausible positive MK estimates was selected on the basis of previous findings [14, 18, 20, 31, 32]. The preliminary MK estimations in our subjects also contributed to selecting this cutoff, as MK values > 2.5 were only rarely observed as outliers.

For subjective image quality assessment, each DTI and DKI map (FA, MD, AD, RD, MK and ADC) were classified by each reader using an adapted Likert scale from 1 (worst) to 5 (best), for the following parameters: anatomical delineation, image graininess, distortion artifacts, motion artifacts (Table 2). Ghost artifacts were also searched for but were not evident in our images, and were thus excluded from this analysis.

**Table 2:** Classification scale for subjective image quality assessment by two readers

Quality parameters	Classification scale				
	1	2	3	4	5
Anatomical delineation	Poor	Suboptimal	Acceptable	Good	Excellent
Image graininess	Unreadable	Detrimental	Not impactful	Mild	Not perceived
Distortion artifacts	Unreadable	Detrimental	Not impactful	Mild	Not perceived
Motion artifacts	Unreadable	Detrimental	Not impactful	Mild	Not perceived

Image graininess and artifacts were classified according to their potential impact on diagnosis, if: rendering the images unreadable, detrimental to diagnosis, present but not impactful, mild, or not perceived

### Statistical analysis

IBM SPSS^®^ statistics v.24 was used for statistical analyses. The Kolmogorov-Smirnov was used to assess the normality of distribution of continuous variables, revealing not all tested variables had a normal distribution. We thus opted for non-parametric tests: Mann-Whitney’s U test was used to compare groups of continuous and ordinal variables; Fisher’s exact test was used for comparing categorical variables.

Bland-Altman analysis was used to assess intra-subject repeatability, and the coefficient of repeatability (r) was calculated according to [33, 34]:

$$\:r=1.96*\sqrt{\raisebox{1ex}{$\sum\:{d}^{2}$}\!\left/\:\!\raisebox{-1ex}{$n$}\right.}$$

Where d is the difference between acquisitions and n the number of volunteers.

Intraclass correlation coefficient was used to assess inter-reader agreement for pancreatic quantification in each map, as follows: <0.5 considered poor, 0.5 to 0.75 considered moderate, 0.75 to 0.9 considered good, > 0.90 considered excellent [35]. For subjective image quality comparisons, weighted Kappa was used to assess inter-reader agreement (quadratic form for weighting difference magnitude), and was classified as: less than chance (< 0), slight (0.01–0.2), fair (0.21–0.4), moderate (0.41–0.6), substantial (0.61–0.8), almost perfect (> 0.81) [36, 37].

*p* < 0.05 was considered significant.

## Results

### Scan length and image quality

Twelve healthy volunteers were included in each group of different MRI protocols (Table 3). Scan length was shorter for the 6 diffusion directions protocol: median 7 min for the full DTI and DKI acquisition (vs. 13 min for 16 directions), and 4 min for DTI only acquisition (vs. 7.5 min for 16 directions).

**Table 3:** Volunteer and scanning characteristics

	16 diffusion directions	6 diffusion directions	*P* value
Number of volunteers	12	12	-
Age (years)	35 (29–60)	37.5 (23–50)	0.443
Sex	8 Female / 4 Male	9 Female / 3 Male	1.000
Full scan length (min)	13 (12–26)	7 (6–10)	< 0.001*
DTI only length (min)	7.5 (6–15)	4 (3–5)	< 0.001*

Results displayed as medians and ranges (minimum-maximum). Mann-Whitney U test was used for comparisons between groups for continuous variables; Fisher’s exact test used for categorical variables. *denotes statistically significant values

**Fig. 1:**
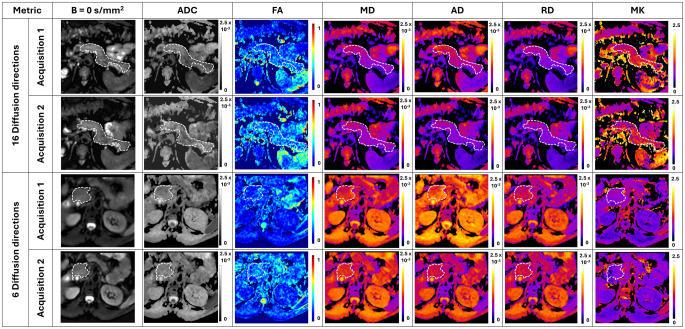
DWI, DTI and DKI maps in two volunteers, for each MRI protocol (6 vs. 16 diffusion directions). Examples show both acquisitions for each volunteer. The pancreas is delimited in white in both volunteers, showing mostly homogeneous values in all maps, with a similar visual quality as the clinical standard ADC maps. MD, AD, RD and ADC maps are expressed in mm^2^/s

Qualitatively, both MRI protocols provided DTI and MK maps with good quality, comparable to the clinical standard ADC maps, as shown in Fig. 1. The subjective evaluation of quality parameters revealed good to excellent anatomical delineation for MD, AD, RD and ADC maps, which was worse for FA and MK maps with mostly acceptable to good classifications (Table 4). Image graininess was classified as mild to not perceived for most MD, AD, RD and ACD maps, while it was more noticeable in FA and MK maps (not impactful to mild). Regarding distortion and motion artifacts, these were more frequently mild for all maps. There were no significant differences between diffusion direction protocols for the quality parameters assessed, with the sole exception of distortion artifacts for MD maps for reader 1 (mild for 6 diffusion directions and not perceived for 16 diffusion directions).

**Table 4:** Subjective image quality assessment by two readers, protocol comparison

DTI/DKI maps	Quality parameters	Reader 1			Reader 2		
		16 diffusion directions	6 diffusion directions	P	16 diffusion directions	6 diffusion directions	P
FA	Anatomical delineation	3.5 (2.0–5.0)	4.0 (2.5–5.0)	0.219	3.25 (2.0–5.0)	3.5 (2.0–5.0)	0.178
	Image graininess	3.00 (2.00–4.0)	4.00 (2.50–4.0)	0.178	3.75 (2.0–4.0)	4.0 (2.0–5.0)	0.242
	Distortion artifacts	4.25 (3.0–5.0)	3.5 (2.0–5.0)	0.198	3.75 (3.0–5.0)	3.5 (2.0–5.0)	0.755
	Motion artifacts	4.0 (3.0–5.0)	4.0 (3.00–5.0)	0.551	4.0 (2.5–5.0)	5.0 (3.0–5.0)	0.590
MD	Anatomical delineation	5.0 (4.0–5.0)	4.5 (3.0–5.0)	0.198	5.0 (4.0–5.0)	5.0 (3.0–5.0)	1.000
	Image graininess	5.0 (4.0–5.0)	5.0 (4.0–5.0)	0.713	5.0 (4.0–5.0)	5.0 (4.0–5.0)	0.478
	Distortion artifacts	5.0 (3.5–5.0)	4.0 (3.0–5.0)	0.01*	4.0 (3.5–5.0)	3.75 (3.0–5.0)	0.06
	Motion artifacts	4.5 (3.0–5.0)	4.0 (4.0–5.0)	0.443	4.5 (3.0–5.0)	4.25 (4.0–5.0)	0.887
AD	Anatomical delineation	5.0 (3.5–5.0)	4.0 (3.5–5.0)	0.068	5.0 (4.0–5.0)	5.0 (3.0–5.0)	0.932
	Image graininess	4.5 (3.5–4.5)	4.0 (3.0–5.0)	0.514	4.75 (3.5–5.0)	5.0 (4.0–5.0)	0.671
	Distortion artifacts	4.75 (4.0–5.0)	4.0 (3.0–5.0)	0.291	4.0 (3.0–5.0)	4.0 (3.0–5.0)	0.319
	Motion artifacts	4.0 (3.5–5.0)	4.75 (4.0–5.0)	0.114	4.0 (3.0–5.0)	4.5 (4.0–5.0)	0.590
RD	Anatomical delineation	5.0 (4.0–5.0)	4.0 (3.5–5.0)	0.078	5.0 (4.0–5.0)	5.0 (3.5–5.0)	0.713
	Image graininess	4.5 (4.0–5.0)	4.25 (4.0–5.0)	0.551	4.75 (3.0–5.0)	5.0 (3.0–5.0)	0.755
	Distortion artifacts	4.5 (4.0–5.0)	4.0 (3.0–5.0)	0.319	4.0 (3.0–5.0)	3.75 (3.0–5.0)	0.219
	Motion artifacts	4.0 (3.5–5.0)	4.75 (4.0–5.0)	0.291	4.5 (4.0–5.0)	4.25 (4.0–5.0)	0.843
MK	Anatomical delineation	3.5 (2.5–5.0)	3.75 (3.0–5.0)	0.887	4.0 (3.0–5.0)	3.5 (3.0–5.0)	0.198
	Image graininess	3.75 (3.0–4.0)	3.5 (3.0–4.5)	0.266	4.0 (3.0–5.0)	3.25 (3.0–4.5)	0.291
	Distortion artifacts	4.5 (3.0–5.0)	4.0 (3.0–5.0)	0.101	4.0 (3.0–5.0)	4.0 (3.0–5.0)	0.843
	Motion artifacts	4.0 (3.0–5.0)	4.0 (4.0–5.0)	0.443	4.0 (3.5–5.0)	4.25 (4.0–5.0)	0.478
ADC	Anatomical delineation	4.0 (3.0–5.0)	4.0 (3.0–5.0)	1.00	4.0 (3.5–5.0)	4.0 (3.0–5.0)	0.977
	Image graininess	4.0 (3.0–4.5)	4.0 (3.0–5.0)	0.843	4.0 (4.0–5.0)	4.5 (3.0–5.0)	0.347
	Distortion artifacts	4.0 (3.0–5.0)	4.0 (3.0–5.0)	0.887	4.0 (3.0– 5.0)	4.0 (3.0–4.5)	0.443
	Motion artifacts	4.0 (3.5–5.0)	4.5 (4.0–5.0)	0.143	4.0 (3.5–5.0)	5.0 (4.0–5.0)	0.068

Median values of classification for each reader for all acquisitions, with averaged repeated acquisitions per subject (12 classifications per reader for each diffusion directions protocol), are presented with minimum-maximum ranges. Mann-Whitney U test is used for comparisons. * Denotes statistically significant results

When assessing inter-reader agreement for image quality classification, weighted Kappa values revealed most often a substantial agreement (2/48 slight, 12/48 fair, 11/48 moderate, 17/48 substantial, 6/48 almost perfect) (Supplementary Table 1). Weighted Kappa values ranged between 0.11 (AD, Image graininess, 6 diffusion directions) and 1.00 (MD, Motion artifacts, 16 diffusion directions).

Regarding the quantification of implausible estimates, these were only relevant in MK maps, having excluded medians of 17.723% of pixels for the 16 diffusion directions protocol (ranging up to 45.3%) and 15.956% of pixels for 6 diffusion directions protocol (ranging up to 41.9%) (*p* = 0.887, Supplementary Table 2).

### Pancreatic DTI and DKI - quantitative analysis

The DTI (FA, MD, AD, RD), MK and ADC measurements extracted from the segmented whole pancreas from all volunteers can be found in Table 5. No differences in measurements were seen for both MRI protocols, except for FA: 0.231 for the 16 directions protocol and 0.276 for the 6 directions protocol (*p* = 0.008). The analysis of the 90th and 10th percentiles also revealed that differences in measurements are limited to FA, while all other DTI and DKI metrics provided similar results for both acquisition protocols.

**Table 5:** DTI, DKI and ADC pancreatic measurements obtained from protocols with 16 and 6 diffusion directions

Metric	16 diffusion directions	6 diffusion directions	*P* value
FA (median)	0.231 (IQR = 0.025)	0.276 (IQR = 0.053)	0.008*
FA (90th P)	0.351 (IQR = 0.060)	0.417 (IQR = 0.095)	0.017*
FA (10th P)	0.134 (IQR = 0.017)	0.165 (IQR = 0.039)	0.006*
MD (median)	0.987 (IQR = 0.277)	1.064 (IQR = 0.232)	0.590
MD (90th P)	1.157 (IQR = 0.313)	1.122 (IQR = 0.262)	0.590
MD (10th P)	0.862 (IQR = 0.200)	0.871 (IQR = 0.224)	0.514
AD (median)	1.203 (IQR = 0.313)	1.343 (IQR = 0.211)	0.219
AD (90th P)	1.407 (IQR = 0.367)	1.5909 (IQR = 0.200)	0.114
AD (10th P)	1.040 (IQR = 0.269)	1.072 (IQR = 0.225)	0.378
RD (median)	0.892 (IQR = 0.250)	0.903 (IQR = 0.218)	0.932
RD (90th P)	1.057 (IQR = 0.297)	1.080 (IQR = 0.252)	0.977
RD (10th P)	0.739 (IQR = 0.183)	0.679 (IQR = 0.210)	0.843
MK (median)	0.663 (IQR = 0.214)	0.647 (IQR = 0.228)	0.843
MK (90th P)	0.839 (IQR = 0.299)	0.822 (IQR = 0.236)	0.671
MK (10th P)	0.338 (IQR = 0.197)	0.356 (IQR = 0.180)	1.000
ADC (median)	1.179 (IQR = 0.228)	1.191 (IQR = 0.152)	0.671
ADC (90th P)	1.446 (IQR = 0.249)	1.430 (IQR = 0.097)	0.887
ADC (10th P)	0.904 (IQR = 0.286)	0.962 (IQR = 0.232)	0.843

Results from pancreatic segmentation from one reader, averaged between two acquisitions for each volunteer. MD, AD, RD and ADC are expressed as x10^− 3^ mm^2^/s to facilitate comparisons. 90th P and 10th P represent 90th and 10th percentiles, respectively. Mann-Whitney U test was used for comparisons between groups. *denotes statistically significant values

Supplementary Figs. 1–4 show the individual subject boxplots for all diffusion measurements in the pancreas, for each acquisition protocol and for each reader. These showcase the consistency of distribution of measured pancreatic values in each map in a subject-by-subject basis, quite similar to the clinical standard ADC; however with MK measurements from some participants displaying large outlier numbers for both readers.

### Repeatability and inter-reader agreement

**Fig. 2:**
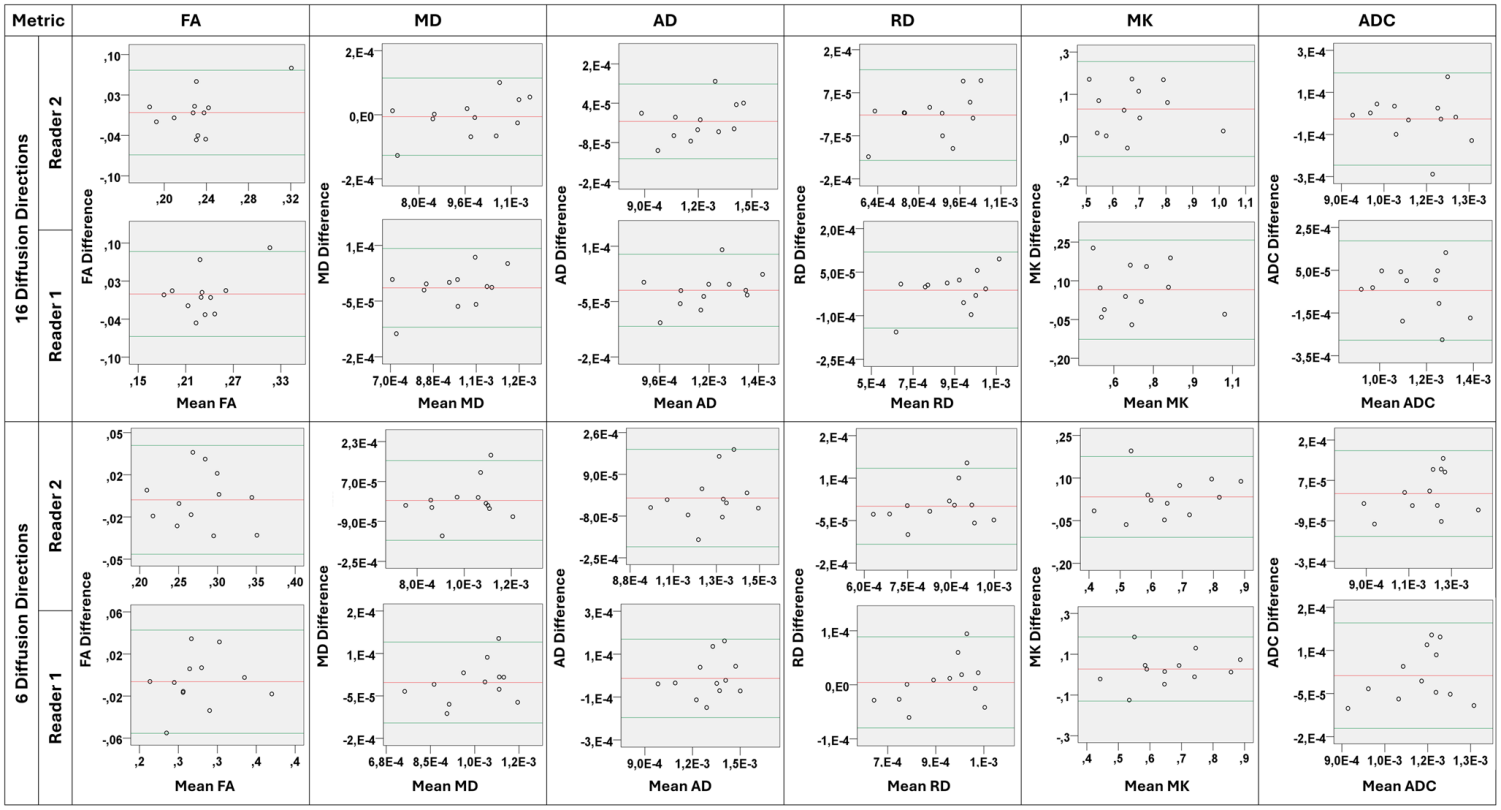
Bland-Altman plots for all volunteers in each MRI protocol and for each reader. The quantitative differences between the first and second acquisition for each subject fall within short 95% confidence intervals (green lines) of their mean (red lines), including zero and presenting only rare and small-magnitude outliers, determining intra-subject repeatability

When assessing intra-subject repeatability, Bland-Altman plots were used to demonstrate measurable differences between the first and second acquisitions for each volunteer and search for proportional bias (Fig. 2). Differences in measurements for all maps fell within short 95% confidence intervals including zero, with only some rare and small-magnitude outliers, determining intra-subject repeatability and no proportional bias. The obtained coefficients of repeatability further determine robustness of DTI with small negligible values across metrics for both protocols (Table 6). However, higher coefficients of repeatability were obtained for MK, especially with the 16-directions protocol, reaching 0.445 (66.9% larger than the median) for the 16 directions and 0.228 (35.2% larger than the median) for the 6 directions protocols, respectively.

**Table 6:** Repeatability coefficients of DTI, DKI and DWI metrics for two readers, for both acquisition protocols, with corresponding proportion relative to the median values

Metric	16 Diffusion Directions		6 Diffusion Directions	
	Reader 1	Reader 2	Reader 1	Reader 2
FA	0.012 (5.2%)	0.053 (23.0%)	0.015 (5.4%)	0.043 (16.2%)
MD	0.040 (4.1%)	0.098 (10.0%)	0.040 (3.8%)	0.018 (1.7%)
AD	0.105 (8.7%)	0.132 (11.1%)	0.046 (3.4%)	0.089 (6.6%)
RD	0.001 (0.1%)	0.083 (9.3%)	0.005 (0.6%)	0.045 (4.9%)
MK	0.326 (49.2%)	0.445 (66.9%)	0.228 (35.2%)	0.186 (28.8%)
ADC	0.180 (15.3%)	0.304 (26.0%)	0.1231 (10.3%)	0.083 (6.9%)

MD, AD RD and ADC are expressed as x10^− 3^ mm^2^/s to facilitate comparisons. The proportion of each coefficient related to its median value is expressed as percentage, to facilitate interpretation

Inter-reader agreement for pancreatic DTI/DKI maps quantitative results after segmentation was excellent for every map and for both MRI protocols (intraclass correlation coefficients > 0.9), as shown in Supplementary Table 1.

## Discussion

This study demonstrates the feasibility, repeatability and inter-reader agreement of pancreatic DTI and DKI, while also providing the expected normal values of the respective maps when performed in the healthy pancreas and a subjective image quality analysis. The two MRI protocols compared showed no significant differences regarding image quality and repeatability, favoring the protocol with 6 diffusion directions due to shorter scan length. Slightly higher FA measurements may however be expected with the shorter protocol.

Nissan et al. have previously demonstrated the feasibility of DTI of the pancreas in healthy volunteers and in patients with pancreatic cancer [16]. Our study further establishes the feasibility of DTI for the pancreas, including data on the intra- and inter-subject repeatability, inter-reader agreement, and different acquisition protocols. Furthermore, our subjective image quality analysis demonstrates DTI and DKI as comparable with the clinical standard ADC, with acceptable to excellent anatomical delineation, not impactful to mild image graininess and mostly mild distortion and motion artifacts.

Our results regarding ADC values are comparable with previous publications, some of which reported variability in ADC measurements with age and gender dependency [38, 39]. Our small sample of volunteers did not allow a comprehensive evaluation of possible age and gender differences influencing measurements in pancreatic DTI/DKI. Nevertheless, previous research did not report gender differences for pancreatic DTI [16].

The use of different numbers of diffusion directions for DTI has been studied in neuroimaging applications, and smaller numbers have been associated with inaccurate measurements, especially affecting FA [40–42]. This was also apparent in our study, where the 6-direction protocol provided higher FA values. Although the absolute difference was not large (0.231 for 16 directions and 0.276 for 6 directions), caution should still be employed when comparing FA values, especially if comparing measurements obtained from different gradient schemes. Other than FA, the remaining DTI and MK maps provided similar values for both direction protocols.

Nissan et al. reported pancreatic FA values of  0.38 ± 0.06 in healthy volunteers, using 30 diffusion directions and a highest b value of 500 s/mm^2^ [16]. Another study investigating DTI for characterizing acute pancreatitis reported FA values of 0.54 ± 0.12 in healthy volunteers, using 9 diffusion directions and highest b value of 500 s/mm^2^ [17]. Our results show lower values for both acquisition protocols, using a highest b value of 1000 s/mm^2^ (0.231 for 16 directions and 0.276 for 6 directions). Factors other than number of diffusion directions, such as signal-to-noise ratio (SNR), denoising techniques and choice of b value may account for disparities in FA measurements, with higher SNR and higher b values both contributing to lower FA estimations [43, 44].

Our results regarding AD measurements provided lower values when compared with a previous study: 1.203E-3 mm^2^/s (IQR = 0.313E-3) and 1.343E-3 mm^2^/s (IQR = 0.211E-3) mm^2^/s respectively for each protocol, while previously reported values were 2.70E-3 ± 0.3E-3 mm^2^/s (using b values of 0 and 500 s/mm^2^) and 2.09E-3 ± 0.25E-3 mm^2^/s (using b values of 100 and 500 s/mm^2^) [16]. The same study reported MD values of 2.04E-3 ± 0.28E-3 mm^2^/s (using b values of 0 and 500 s/mm^2^) and 1.44E-3 ± 0.16E-3 mm^2^/s (using b values of 100 and 500 s/mm^2^), while our results provided values of 0.987E-3 mm^2^/s (IQR = 0.277E-3) and 1.064E-3 mm^2^/s (IQR = 0.232E-3) respectively for each protocol. Although our measurements for AD and MD are lower, they are aligned with the conclusions of this study, where using a lower b value ≥ 100 s/mm^2^ for removing perfusion effects provides lower estimations of diffusion coefficients. Our choice of higher b value also probably contributed to the lower diffusivity estimations [44]. These findings prompt caution when comparing diffusivity values between patients, as DTI parameters should be maintained as equal as possible to provide comparable estimates.

Noda et al. evaluated pancreatic DKI in diabetic patients as a potential biomarker for HbA1C concentration levels, with a 3 diffusion directions protocol with b values of 0, 100, 500, 1000, 1500, and 2000 s/mm^2^ [20]. They described a positive correlation between MK and HbA1C, and reported MK values of 0.60 ± 0.06 for patients with HbA1c < 5.7%, the group most comparable to our healthy volunteers. Despite differences in acquisition protocols, our results are just in line with these, with MK values of 0.663 (IQR = 0.214) and 0.647 (IQR = 0.228) respectively for each protocol. Granata et al. assessed DKI in patients with pancreatic cancer for its diagnosis, with a maximum b value of 1000 s/mm^2^ [18]. They reported kurtosis-corrected diffusion coefficient (D) as a valuable diagnostic measurement, but not MK. These results are encouraging for DKI as a way to get more accurate diffusivity estimates for pancreatic cancer diagnosis. While Granata et al. did not show that MK has diagnostic value, future studies using higher b values (which are more adequate for DKI) might provide better results for MK [15].

Regarding scan length, the shorter 6-directions protocol may provide a valuable advantage for clinical imaging. Shorter acquisitions, besides being better tolerated by patients, are less affected by motion artefacts [45, 46]. Shorter scan lengths are also obviously desirable in any Radiology department allowing for higher throughputs.

When assessing the number of excluded voxels due to implausible values, DTI maps for both protocols were mostly unaffected. However, MK maps were clearly affected, with median 17.723% (16 directions protocol) and 15.956% (6 directions protocol) excluded pixels. Furthermore, high coefficients of repeatability obtained from MK measurements, especially with the 16-directions protocol (reaching 66.9% relative to its median value for one of the readers), indicate important variability in MK estimates. Kurtosis estimates are known to be more sensitive to image noise and artefacts than their diffusion counterparts, which could explain these results [47, 48]. Although denoising and artifact correction algorithms were used in pre-processing, and the MK estimation was performed with a robust signal-average technique, these results still advise caution when using DKI for pancreatic imaging [29, 47].

Possible causes for such inaccurate MK estimations might include physiological cardiovascular and bowel motion, and bowel gas artifacts, which were not directly addressed in our study and present relevant points for evaluation in future studies [49–51]. Other possible contributors to inaccurate MK estimations might be the parallel acquisition induced artifacts, such as ghosting and low SNR, which were not obvious in our images [52]. However, due to the reduced field of view, areas outside the body where ghosting artifacts are more noticeable were not evaluated. Furthermore, methods for improving SNR, such as increasing slice thickness or signal averages may be used to improve MK estimations’ accuracy, however at the cost of lower spatial resolution and lengthier acquisitions, respectively. In this study, we used a slice thickness of 3 mm to achieve higher spatial resolution and reduce partial volume effects. However, this decision resulted in lower SNR compared to a standard 5 mm thickness, which is a known trade-off [53]. The impact of slice thickness on SNR can be quantified theoretically: assuming all other imaging parameters remain constant (e.g., in-plane resolution, signal averages, receiver bandwidth, and diffusion directions), SNR is proportional to slice thickness. Therefore, increasing the slice thickness from 3 mm to 5 mm would lead to an expected SNR gain of 5/3. Applying uncertainty propagation to the DKI model, the standard deviation of MK estimates scales inversely with SNR [54]. This means that increasing the slice thickness from 3 mm to 5 mm would theoretically reduce the MK estimation error by a factor of 3/5, corresponding to a 40% reduction in the repeatability coefficient of MK estimation. These theoretical predictions provide a framework for generalizing our results to different slice thicknesses.

This study has limitations. First, the sample group of volunteers can be considered small, precluding analyses on possible gender and age differences. Second, our sample included mostly young and active people, which might not provide an optimal reference for subjects most often affected by pancreatic disease, later in life. Third, the volunteers’ body-mass index was not registered, and could not be correlated with our results, as a possible source of bias. Fourth, as mentioned above, motion artifacts correction was not directly implemented in our study and presents an important point for improvement in future research. Fifth, our ADC computing method used the first direction of the DTI data, which differs from the current clinical standard trace of three orthogonal axes, and may entail diffusion anisotropy susceptibility. Finally, our pancreatic segmentation did not allow separate analyses on organ parts (head, body and tail), which have previously been described to have slightly decreasing diffusivities [16]. This remains an interesting point for further research.

In conclusion, DTI and DKI of the pancreas are feasible and repeatable techniques in a clinical 3T scanner, with excellent inter-observer agreement. However, issues with MK inaccuracies should be kept in mind, and further research might provide improvements by correcting sources of artifacts. A shorter protocol with 6 diffusion directions may be preferred, as it performs mostly similarly to a longer 16 directions protocol.

## Electronic supplementary material

Below is the link to the electronic supplementary material.

Supplementary Material 1:

Supplementary Material 2:

Supplementary Material 3:

Supplementary Material 4:

Supplementary Material 5

Supplementary Material 6

## Data Availability

The datasets generated and/or analyzed during the current study are not publicly available, but are available from the corresponding author on reasonable request. Any human-derived data will be completely anonymized.

## Abbreviations

AD: Axial diffusivity
ADC: Apparent diffusion coefficient
CT: Computerized tomography
DKI: diffusion kurtosis imaging
DTI: Diffusion tensor imaging
DWI: Diffusion weighted imaging
FA: Fractional anisotropy
ICC: Intraclass correlation coefficient
MD: Mean diffusivity
MK: Mean kurtosis
MRCP: Magnetic resonance cholangiopancreatography
PDAC: Pancreatic ductal adenocarcinoma
RD: Radial diffusivity
SB-IPMN: Side branch intraductal papillary mucinous neoplasia
SE-EPI: Spin-echo echo planar imaging
SNR: Signal to noise ratio
TSE: Turbo spin-echo
