# Supplementary figures and images for: Diffusion tensor imaging and diffusion kurtosis imaging of the pancreas - feasibility, robustness and protocol comparison in a healthy population
Source: Abdom Radiol (NY). 2025 Mar 26;50(10):4563–74. doi: 10.1007/s00261-025-04889-w (PMC12454463; doi:10.1007/s00261-025-04889-w)

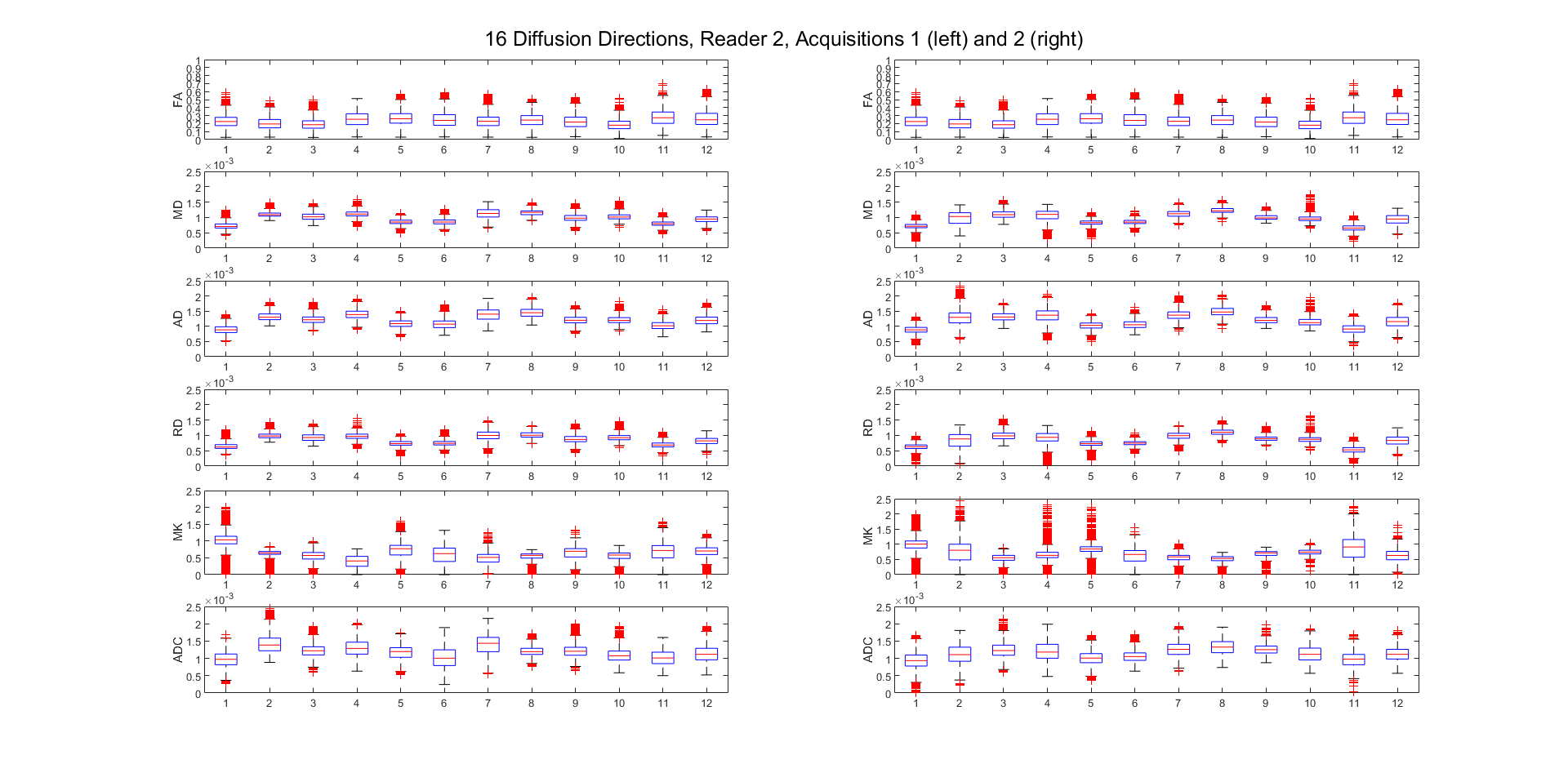

Supplement: Supplementary file 1 — Supplementary Material 1: [file 261_2025_4889_MOESM1_ESM.tif]

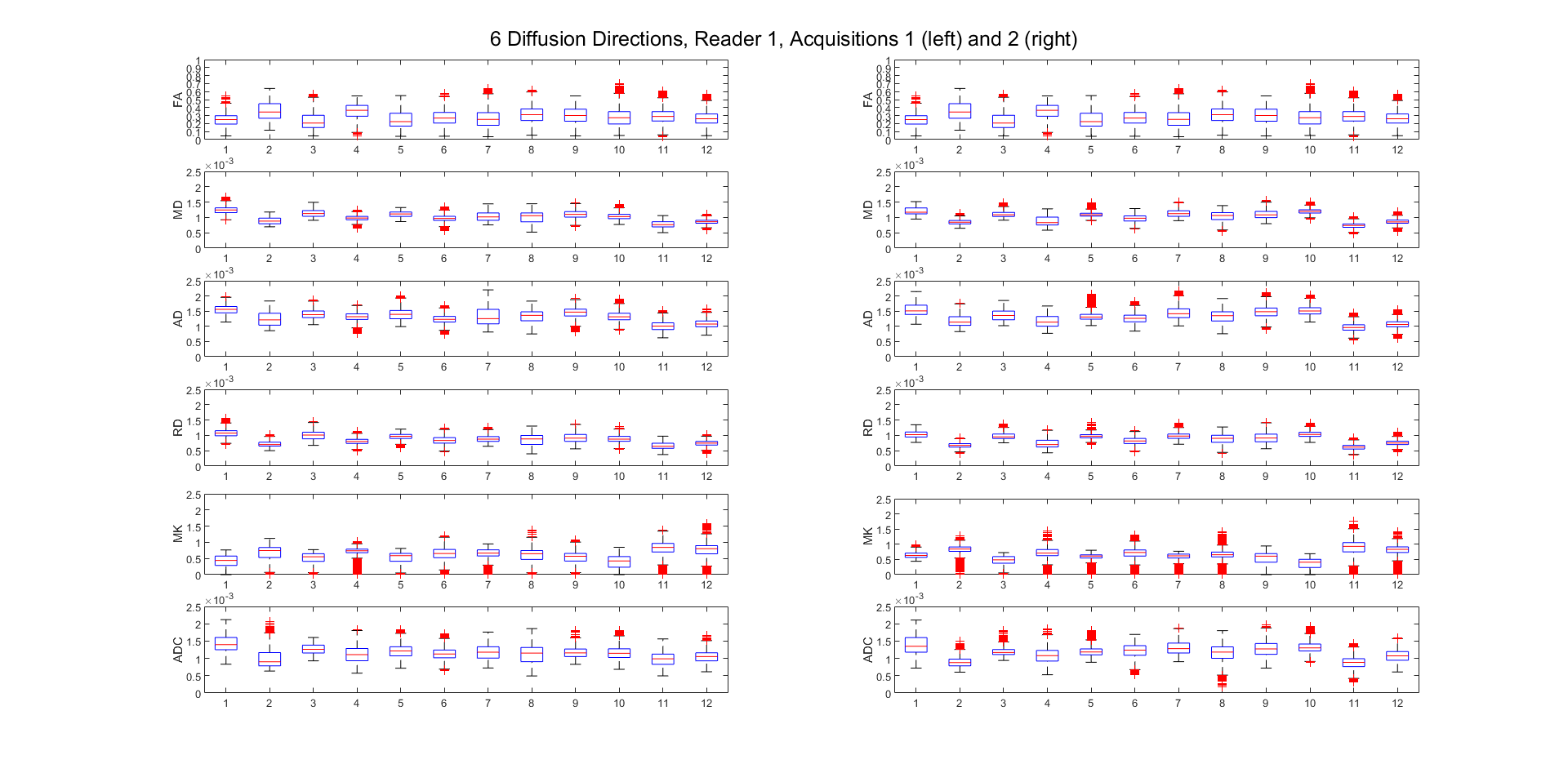

Supplement: Supplementary file 2 — Supplementary Material 2: [file 261_2025_4889_MOESM2_ESM.tif]

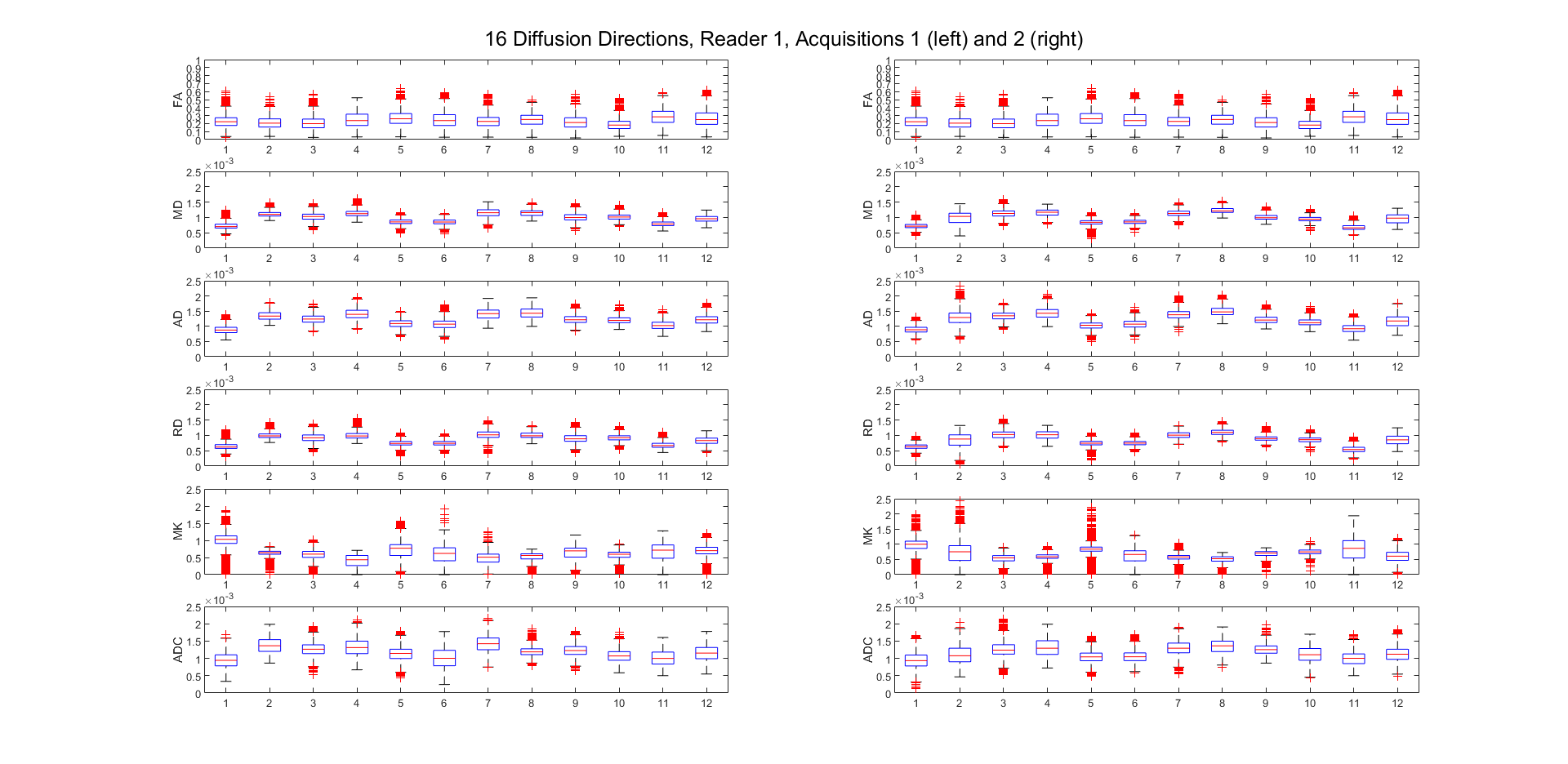

Supplement: Supplementary file 3 — Supplementary Material 3: [file 261_2025_4889_MOESM3_ESM.tif]

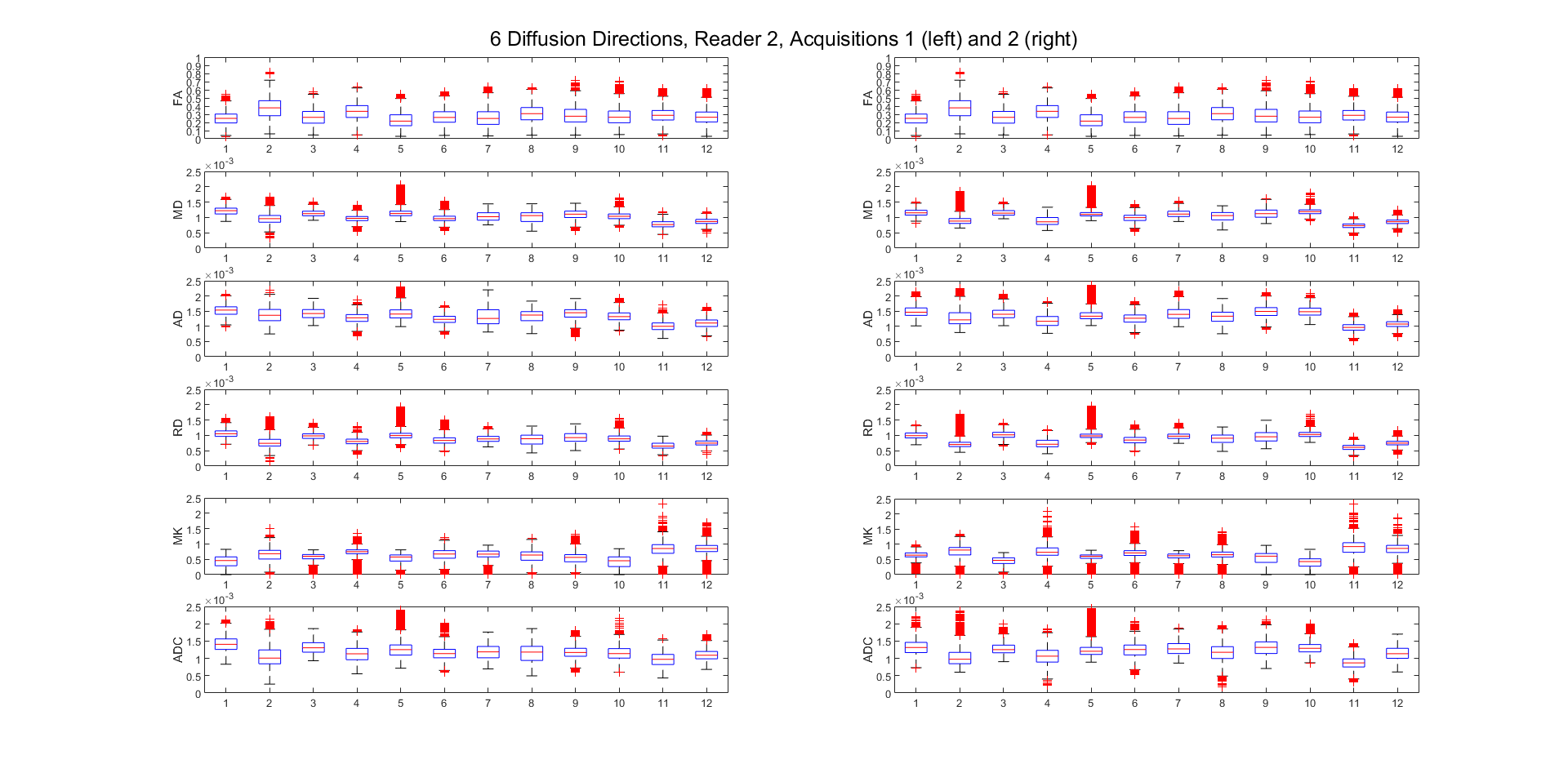

Supplement: Supplementary file 4 — Supplementary Material 4: [file 261_2025_4889_MOESM4_ESM.tif]
