# Supplementary material for: Diffusion tensor imaging and diffusion kurtosis imaging of the pancreas - feasibility, robustness and protocol comparison in a healthy population
Source: Abdom Radiol (NY). 2025 Mar 26;50(10):4563–74. doi: 10.1007/s00261-025-04889-w (PMC12454463; doi:10.1007/s00261-025-04889-w)
Supplement: Supplementary file 5 — Supplementary Material 5 [file 261_2025_4889_MOESM5_ESM.docx]

Supplementary Table 1. Subjective image quality assessment by two readers, inter-reader reliability analysis.

| **DTI/DKI maps** | **Quality parameters** | **16 diffusion directions** | | | **6 diffusion directions** | | |
| --- | --- | --- | --- | --- | --- | --- | --- |
|  |  | Reader 1 | Reader 2 | Weighted Kappa | Reader 1 | Reader 2 | Weighted Kappa |
| **FA** | **Anatomical delineation** | 3.33  (2 – 5) | 3.21  (2 – 5) | 0.73  (0.57 – 0.88) | 3.83  (2 - 5) | 3.67  (2 – 5) | 0.64  (0.39 – 0.89) |
|  | **Image graininess** | 3.13  (2 – 4) | 3.21  (2 – 4) | 0.61  (0.40 – 0.82) | 3.46  (2 – 4) | 3.71  (2 – 5) | 0.65  (0.48 – 0.81) |
|  | **Distortion artifacts** | 4.17  (3 – 5) | 3.75  (3 – 5) | 0.67  (0.50 – 0.84) | 3.71  (2 – 5) | 3.63  (2 – 5) | 0.90  (0.80 – 1.00) |
|  | **Motion artifacts** | 4.08  (3 – 5) | 4.04  (2 – 5) | 0.88  (0.86 – 1.00) | 3.96  (3 – 5) | 4.25  (3 – 5) | 0.23  (-0.07 – 0.53) |
| **MD** | **Anatomical delineation** | 4.79  (4 – 5) | 4.79  (4 – 5) | 0.50  (0.07 – 0.92) | 4.50  (3 – 5) | 4.67  (3 – 5) | 0.80  (0.58 – 1.00) |
|  | **Image graininess** | 4.83  (4 – 5) | 4.79  (4 – 5) | 0.86  (0.60 – 1.00) | 4.75  (4 – 5) | 4.63  (4 - 5) | 0.52  (0.17 – 0.88) |
|  | **Distortion artifacts** | 4.58  (3 – 5) | 4.25  (3 – 5) | 0.47  (0.17 – 0.78) | 3.83  (3 – 5) | 3.75  (3 – 5) | 0.90  (0.76 – 1.00) |
|  | **Motion artifacts** | 4.42  (3 – 5) | 4.42  (3 – 5) | 1.00  (1.00 – 1.00) | 4.25  (4 – 5) | 4.42  (4 – 5) | 0.46  (0.11 – 0.80) |
| **AD** | **Anatomical delineation** | 4.67  (3 – 5) | 4.71  (3 – 5) | 0.79  (0.52 – 1.00) | 4.21  (3 – 5) | 4.63  (3 – 5) | 0.15  (-0.08 – 0.37) |
|  | **Image graininess** | 4.17  (3 – 5) | 4.58  (3 – 5) | 0.40  (0.09 – 0.71) | 4.04  (3 – 5) | 4.67  (4 – 5) | 0.11  (-0.14 – 0.36) |
|  | **Distortion artifacts** | 4.58  (4 – 5) | 4.13  (4 – 5) | 0.37  (0.17 – 0.57) | 4.13  (3 – 5) | 3.83  (3 – 5) | 0.34  (-0.02 – 0.70) |
|  | **Motion artifacts** | 4.17  (3 – 5) | 4.29  (3 – 5) | 0.73  (0.50 – 0.96) | 4.33  (4 – 5) | 4.42  (4 – 5) | 0.75  (0.50 – 1.00) |
| **RD** | **Anatomical delineation** | 4.75  (4 – 5) | 4.63  (4 – 5) | 0.71  (0.42 – 1.00) | 4.33  (3 – 5) | 4.67  (3 – 5) | 0.28  (-0.12 – 0.67) |
|  | **Image graininess** | 4.58  (4 – 5) | 4.46  (3 – 5) | 0.44  (0.17 – 0.72) | 4.46  (3 - 5) | 4.54  (3 – 5) | 0.13  (-0.26 – 0.52) |
|  | **Distortion artifacts** | 4.50  (4 – 5) | 4.13  (3 – 5) | 0.44  (0.22 – 0.66) | 4.13  (3 - 5) | 3.79  (3 – 5) | 0.28  (-0.09 – 0.65) |
|  | **Motion artifacts** | 4.54  (3 – 5) | 4.42  (4 – 5) | 0.56  (0.28 – 0.83) | 4.33  (4 – 5) | 4.42  (4 – 5) | 0.43  (0.08 – 0-77) |
| **MK** | **Anatomical delineation** | 3.67  (2 – 5) | 4.08  (2 – 5) | 0.63  (0.39 – 0.86) | 3.63  (3 – 5) | 3.71  (3 – 5) | 0.74  (0.53 – 0.94) |
|  | **Image graininess** | 3.71  (3 – 5) | 3.79  (3 – 5) | 0.18  (-0.18 – 0.64) | 3.50  (3 – 5) | 3.50  (3 – 5) | 0.89  (0.75 – 1.00) |
|  | **Distortion artifacts** | 4.38  (3 – 5) | 3.96  (3 – 5) | 0.52  (0.25 – 0.79) | 3.88  (3 – 5) | 3.88  (3 – 5) | 0.40  (0.04 – 0.75) |
|  | **Motion artifacts** | 4.08  (3 – 5) | 4.29  (3 – 5) | 0.72  (0.52 – 0.92) | 4.00  (4 – 5) | 4.42  (4 – 5) | 0.74  (0.48 – 1.00) |
| **ADC** | **Anatomical delineation** | 4.08  (3 – 5) | 4.04  (3 – 5) | 0.67  (0.40 – 0.93) | 4.08  (3 – 5) | 4.21  (3 – 5) | 0.59  (0.34 – 0.85) |
|  | **Image graininess** | 3.96  (3 – 5) | 4.13  (4 – 5) | 0.36  (-0.04 – 0.76) | 3.96  (3 – 5) | 4.38  (3 – 5) | 0.27  (-0.07 – 0.62) |
|  | **Distortion artifacts** | 4.08  (3 – 5) | 3.96  (3 – 5) | 0.73  (0.45 – 1.00) | 4.04  (3 – 5) | 3.71  (3 – 5) | 0.23  (-0.07 – 0.53) |
|  | **Motion artifacts** | 4.00  (3 – 5) | 4.25  (3 – 5) | 0.67  (0.34 – 0.99) | 4.29  (4 – 5) | 4.50  (4 – 5) | 0.43  (0.11 – 0.76) |

Average values of classification for each reader for all acquisitions, including repeated acquisitions per subject (24 classifications per reader for each diffusion directions protocol), are presented with minimum-maximum ranges. Weighted Kappa (quadratic) was used to assess inter-observer reliability and is presented with 95% confidence intervals.

Supplementary Table 2. Excluded artifactual voxels in DTI, DKI and ADC maps.

| **Metric** | **16 diffusion directions** | **6 diffusion directions** | **P value** |
| --- | --- | --- | --- |
| **FA** | 0.000% | 0.000% | 0.514 |
| **MD** | 0.000% | 0.000% | 0.514 |
| **AD** | 0.000% | 0.000% | 0.514 |
| **RD** | 0.000% | 0.000% | 0.514 |
| **MK** | 17.723% (IQR = 18.05) | 15.956% (IQR = 9.042) | 0.887 |
| **ADC** | 0.000% | 0.000% | 1.000 |

Excluded voxels are represented in percentages, with median values and IQR. Mann-Whitney U test was used for comparisons between groups.

Supplementary Table 3. Intraclass correlation coefficients of DTI, DKI and DWI metrics for two readers, for both acquisition protocols.

| **Metric** | **ICC** | |
| --- | --- | --- |
|  | **16 Diffusion Directions** | **6 Diffusion Directions** |
| **FA** | 0.967 (0.925-0.985) | 0.910 (0.803-0.960) |
| **MD** | 0.994 (0.986-0.997) | 0.985 (0.965-0.993) |
| **AD** | 0.995 (0.989-0.998) | 0.969 (0.929-0.986) |
| **RD** | 0.990 (0.977-0.996) | 0.986 (0.968-0.994) |
| **MK** | 0.992 (0.982-0.997) | 0.984 (0.963-0.993) |
| **ADC** | 0.971 (0.935-0.988) | 0.962 (0.914-0.983) |

A two-way mixed model of single measures was used.

ICC is displayed with 95% confidence intervals. A p value of <0.001 was observed in all displayed results.
