# Supplementary material for: Diffusion tensor imaging and diffusion kurtosis imaging of the pancreas - feasibility, robustness and protocol comparison in a healthy population
Source: Abdom Radiol (NY). 2025 Mar 26;50(10):4563–74. doi: 10.1007/s00261-025-04889-w (PMC12454463; doi:10.1007/s00261-025-04889-w)
Supplement: Supplementary file 6 — Supplementary Material 6 [file 261_2025_4889_MOESM6_ESM.docx]

Supplementary Figure 1 - Boxplots for the 6 diffusion directions protocol as assessed by Reader 1, for each DTI, DKI and DWI metric (FA, MD, AD, RD, MK, ADC) for each volunteer.

Supplementary Figure 2 - Boxplots for the 16 diffusion directions protocol as assessed by Reader 1, for each DTI, DKI and DWI metric (FA, MD, AD, RD, MK, ADC) for each volunteer.

Supplementary Figure 3 - Boxplots for the 6 diffusion directions protocol as assessed by Reader 2, for each DTI, DKI and DWI metric (FA, MD, AD, RD, MK, ADC) for each volunteer.

Supplementary Figure 4 - Boxplots for the 16 diffusion directions protocol as assessed by Reader 2, for each DTI, DKI and DWI metric (FA, MD, AD, RD, MK, ADC) for each volunteer.
